# REMOTE CONTROL THE PHYSICAL ACTIVITY AS HUMAN HEALTH ASSESSMENT OF FUNCTIONAL AGING

**DOI:** 10.1093/geroni/igad104.2934

**Published:** 2023-12-21

**Authors:** Olena Tomarevska, Oleksandr Poliakov

**Affiliations:** D.F. Chebotarev Instutute of Gerontology of the NAMSU, Kyiv, Kyyiv, Ukraine; D.F. Chebotarev Institute of Gerontology of the NAMS of Ukraine, Kyiv, Kyyiv, Ukraine

## Abstract

The level of physical activity is modified by the functional age of a person in accordance with his chronological age. Most of the evidence comes from the positive impact of physical activity on the musculoskeletal system and cognitive functions, microcirculation and cardiovascular system in the working population and retirees. In the current study, an online method the investigation of physical activity was tested. Functional age was calculated using appropriate indicators according to age standards: systolic and diastolic pressure, standing on the left leg with closed eyes, maximum breath holding time after a deep inhalation, maximum breath holding time after a deep exhalation. The state of health is extrapolated to the need for specialist supervision in 58.96% of older workers. Thus, the study showed the need to determine the functional age of respondents 50 years and older. Thus, a slowed rate of aging observed in 23.5% of cases, physiological - in 18% of cases, accelerated rate of aging - in 58.5% of cases. In the exams 441 persons, age 50-90 years, reliable associative links were observed rate of functional aging with a decrease in physical activity according to the indicators of individual daily mileage (r=-0.273, p< 0.001) and the variability of physical exercises (r=-0.161, p< 0.001). The pace of functional aging and state of health were reliably correlated (r=-0.364, p< 0.001) in individuals, respectively, with the preservation of individual daily mileage and the performance of daily physical exercises. Thus, aerobic activity is an important in maintaining health in terms of functional indicators.

